# A Visual ERP Study of Impulse Inhibition following a Zaleplon-Induced Nap after Sleep Deprivation

**DOI:** 10.1371/journal.pone.0095653

**Published:** 2014-05-07

**Authors:** Qianru Zhang, Yang Liao, Jianlin Qi, Yongqi Zhao, Tianli Zhu, Zhaohui Liu, Xufeng Liu

**Affiliations:** 1 Department of Psychology, Fourth Military Medical University, Xi'an Shaanxi, China; 2 Department of Rehabilitative Physioltherapy of Tangdu Hospital, Fourth Military Medical University, Xi'an Shaanxi, China; 3 Department of Clinical Psychology, Air Force General Hospital, Beijing, China; 4 Institute of Basic Medical Sciences, Academy of Military Medical Science, Beijing, China; University of Pennsylvania, United States of America

## Abstract

The side effects of a zaleplon-induced nap as a countermeasure in the reduction of impulse inhibition function decline following 30 h of sleep deprivation (SD) were examined by event-related brain potentials. Sixteen adult participants performed a Go/NoGo task at five time points: (1) baseline; (2) after 30 h of SD; (3) upon sudden awakening, also called 2 h post-drug; (4) 4 h post-drug; and (5) 6 h post-drug. Behavior results show an increase in both reaction time and false alarm rates after SD and sudden awakening, and a marked decrease at 4 h and 6 h post-drug in zaleplon and placebo conditions. However, no difference was observed between the zaleplon condition and the placebo condition. In event-related potential (ERP) reults compared with results obtained under control conditions, NoGo-P3 latencies significantly increased, whereas the Nogo-P3 amplitude decreased after 30 h of SD and sudden awakening in both the zaleplon condition and the placebo condition. These results indicate that SD attenuates resource allocation and error monitoring for NoGo stimuli. In addition, NoGo-P3 latencies were longer in the zaleplon condition compared with the placebo condition at sudden awakening. Additionally, the NoGo-P3 latencies were shorter in the zaleplon condition than in the placebo condition at 4 h and 6 h post-drug. These results indicate that zaleplon at a dose of 10 mg/day may help subjects achieve a better recovery or maintain better impulse inhibition function, although the side effects of zaleplon last at least 2 h post-drug.

## Introduction

In the current professional environment, it is an inevitable reality that people tend to work for prolonged or continuous hours. According to a survey, more than 20% of adults suffer from some form of sleep deprivation (SD) in modern society [1]. Various categories of professionals such as industrial shift workers, workers in transport and telecommunication sectors, trans-meridian pilots, medical and ancillary staff in hospitals, and armed forces personnel, suffer from sleep deprivation of differing magnitudes [2]. Loss of sleep is especially commonplace in soldiers during wartime and rescue workers in disaster areas following earthquakes, flood, etc. Although equipment may be able to operate for prolonged working hours, personnel are not capable of working for days without proper rest and recovery. When extended hours are a necessity, personnel must attempt to identify a manner in which to maintain good executive functions to fulfill their duties. Therefore, the clarification of the far-reaching effects of sleep loss on executive function and the identification of the corresponding countermeasures from a scientific perspective are of great importance.

Executive function, which means planning, execution and inhibition of an action, is a part of cognitive functions and plays an important part in regulating human behavior. Particularly, Inhibitory control, as an important component of executive function, has attracted a great deal of attention among psychological and medical researchers. Several recent studies observed that sleep deprivation impaired performance on a Go/NoGo task, which is the classic experiment to assess inhibition control [3], [4]. The NoGo stimuli generally elicit a fronto-central, negative–positive complex in the event-related potentials (ERP) that have been labeled as components NoGo-N2 and NoGo-P3, respectively. N2 shows a negative deflection in an interval of 200–300 ms following the imperative stimulus, and P3 reveals a prominent positive deflection in a time window of 300–500 ms after the stimulus is administered. These components are considered indices of different aspects of inhibition that originate in the prefrontal cortex [5], [6], [7], [8], [9], [10], . NoGo-N2 is not in the overt response process [12]; it reflects either a pre-motor inhibition process [7], [9], [13] or the detection of the conflict between concurrent pre-motor action tendencies [11]. Therefore, NoGo-N2 can be relevant to pre-motor performance monitoring and control mechanism. NoGo-P3 has been consistently connected with an inhibitory process when overt motor responses are required because it varies with response priming [10] or with the type of response [12]. According to these results, we could identify the change of sub-processes of inhibitory control by ERP.

Protecting cognitive function from fatigue-related errors is challenging; however, simple strategies exist that can mitigate the damage of sleep deprivation. Readily available stimulants, including some energy foods such as chocolate, coffee and tea; drugs such as modafinil; or a scheduled nap can be effective [14], [15], [16], [17]. Moreover, there is ample evidence indicating that a nap taken during a long period of continuous wakefulness is quite beneficial in improving alertness and performance [18], [19], [20], [21], [22], [23]. When sufficient sleep is not possible, scheduled naps for as little as 30 min can be partially restorative [24]. Unfortunately, staff shortages and work demands render scheduled naps problematic in the real world. It may be impossible to schedule naps at the time personnel require sleep. In addition, the heat, environmental noise, lighting and anxiety present in operational environments may undermine personnel's ability to initiate and maintain effective sleep. Therefore, to provide a manner for personnel to obtain required sleep whenever the chance to sleep occurs, hypnotics such as like zaleplon may be useful.

Zaleplon is a short-duration sleep aid that has been shown to reduce sleep latency in insomniacs at the 10-mg dose level [25]. Zaleplon's orally disintegrating tablets (ODTs) differ from traditional tablets in that they are designed to be dissolved on the tongue rather than swallowed whole. The ODT serves as an alternative dosage form for patients who experience dysphagia (difficulty in swallowing) or for cases in which compliance is a known issue and thus an easier dosage form ensures that medication is taken. Taken orally, zaleplon reaches full concentration in approximately 1 h, and its absorption is rapid. The elimination half-life of zaleplon is 1 h.

In the present study, the ERP method was used to record the change in inhibitory control following a zaleplon- or placebo-induced nap after sleep deprivation. With NoGo-P3 and NoGo-N2 as the indices, we sought to compare the effects of a zaleplon-induced nap with those of a placebo-induced nap as a counter measure in the reduction of inhibitory control following 30 h of SD. We assumed that there would be different effects between zaleplon and the placebo.

## Materials and Methods

### Participants

Sixteen healthy male undergraduates participated in the study; the mean age of the participants was 21.8 years (range 19–23 yrs). According to Annett's Hand Preference Questionnaire, all participants were right-handed, had normal or corrected-to-normal vision, and were free of medical and psychiatric disorders. Based on a participant demographic survey, participants worked regular shifts (i.e., no night-shift workers), and were not heavy caffeine, alcohol or tobacco users. All subjects reported averaging 7–9 h of sleep habitually. According to Owl and Lark's questionnaire[26], [27],subjects did not display “morningness” or “eveningness”. All participants provided written formal consent after having received a detailed explanation of the study procedures and received financial compensation for the inconvenience of participating in each phase of the study. The protocol of the experiment was approved by the Ethics Committee of The Fourth Military Medical University.

### Stimuli and procedure

**Figure 1:**
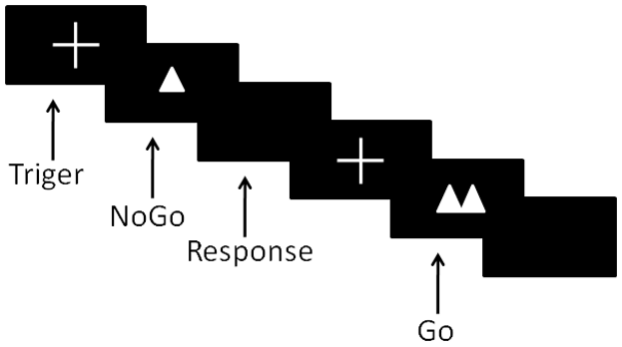
Temporal sequence of events in task trial.

Participants were comfortably seated in an acoustically and electrically shielded room. The distance between the computer screens and the participants was 80 cm. Participants performed a Go/NoGo task to visual stimuli using personal computers. Stimuli were presented individually on a computer screen in white on a black background. The participants had to press a key with the forefinger of their dominant hand after Go-stimuli (two triangles side by side) and refrain from responding after NoGo-stimuli (only one triangle) as fast as possible. Each trial began with a small white cross (+) at the center of the screen against a black background for 100 ms. Then, stimulus occurred for 200 ms in the center of the screen with an inter-stimulus interval of between 1000 ms and 1200 ms randomly as shown in Fig. 1. The task directions emphasized both the speed and accuracy of responding. The task contained 200 stimuli (60% Go and 40% NoGo) in random order. All subjects received a training session to ensure that they understood the task correctly; in the training session, the hit rates reached 98% or above. The stimuli procedure and behavioral data were collected using the Stim-2 software system.

**Figure 2:**
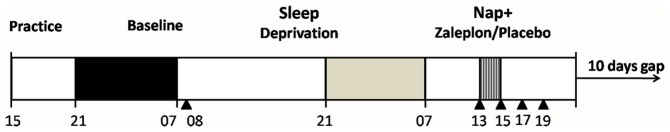
Schematic diagram of experimental design. Subjects stayed inside the laboratory from 15:00 on Day 1 to 19:00 on Day 3. The black area represents a 10 h nocturnal period in bed for sleep (21:00–07:00). The gray area represents a 10 h nocturnal period (21:00–07:00) of sleep deprivation. The subjects actually stayed awake continuously for a total of 30 h during the study. The shadow area represents a 2-h daytime nap period (13:00–15:00). Triangles indicate the 5 administrations of the Go/NoGo task: after 1 h of scheduled wakefulness (Baseline); after 30 h of continuous wakefulness (SD 30 h); after sudden awakening from a nap (Post-drug 2 h); after 2 h of wakefulness from the nap (Post-drug 4 h); and after 4 h of wakefulness from the nap (Post-drug 6 h).

Subjects were in a laboratory for 2 consecutive days and nights. Each participant experienced both of the drug conditions. They experienced the zaleplon condition and the placebo condition by ABBA design, and the washout period lasted 10 days (Fig. 2). The selection of drug dose was 10 mg. The study was fully counterbalanced and double blind. First, subjects received 1 baseline night with 10 h of time in bed for sleep. Then, they were kept wake for 30 h of total sleep deprivation (TSD), which entailed missing the next night of sleep. Before the 30 h of SD, subjects took their first test as a baseline at 8:00 a.m. Finally, they were allowed a 2-h nap; however, before a nap, they received the second test at 1:00 p.m. The members in the zaleplon condition took zaleplon in orally disintegrating tablets when they began to nap whereas the members of the placebo condition just took the placebo. The nap lasted 2 h from 1:00 p.m. to 3:00 p.m. Following a sudden awakening from the daytime nap, the third test was administered. Subjects completed the fourth and fifth tests at 5:00 p.m. and 7:00 p.m., respectively. All participants also performed the test at five different time points without sleep deprivation or any drugs as control condition.

The experiment was conducted in the controlled laboratory environment of the cognitive electrophysiology research center at The Fourth Military Medical University. We supplied two bedrooms and bathrooms. In addition, we provided meals at 07:30, 11:30, and 17:30; water was available at any time. Between performance tests and meals, subjects were permitted only non-vigorous activities. Subjects' behavior was monitored throughout the experiment by trained research assistants. During the experiment, subjects were not allowed to use caffeine, alcohol, tobacco or other drugs influencing sleep quality during the course of the study.

### ERP recording

The continuous scalp electroencephalogram (EEG) was recorded using an electrode cap with electrodes placed at 32 sites of the International 10–20 system. Only midline sites Fz, FCz and Cz are statistically compared here. The electrodes were average mastoids reference (A1 and A2), and the subjects were grounded using an electrode placed on the forehead (FPz). Electrode impedance was kept below 5 kΩ. The sampling rate was set at 1000 Hz. The EEG was amplified by a Neuroscan SynAmps^2^ amplifier with a 0.02 Hz high-pass and 100 Hz low pass. EEG analyses were conducted using the Neuroscan software package (Versions 4.3).

The EEG was segmented into the epoch from 200 ms pre-stimulus to 600 ms post-stimulus, and the baseline was corrected to the mean amplitude of 200 ms before the stimulus. The trials contaminated with artifacts greater than ±100 µV were rejected before averaging. We removed the trials with response times shorter than 100 ms because they were assumed to reflect non-deliberate behavior. ERPs were calculated using correct responses only. The NoGo-P3 amplitudes and latencies at the Fz, FCz, and Cz sites were measured as maximum positive values from time windows of 300–550 ms. The NoGo-N2 amplitudes and latencies at the Fz, FCz, and Cz sites were measured as peak values of the negative component at 200–350 ms post-stimulus intervals.

### Data measurement and analysis

The data were analyzed using SPSS 16.0 for Windows. Values were expressed as the mean ± SD. Analysis of variance (ANOVA) with repeated measures on two factors (condition and time) was used to analyze the data. A Greenhouse-Geisser adjustment was made for variables that failed Mauchly's Test of Sphericity. Bonferroni correction was used in pairwise comparison when appropriate. First, we focused on mean reaction time, hit rates and false alarm rates. In further analyses, amplitudes and latencies of NoGo-N2 and NoGo-P3 examined at electrode Fz, FCz and Cz were analyzed.

## Results

### Behavioral performance

Table 1 shows the means and standard deviations of the mean reaction time, hit rates in the Go trials and false alarm rates in the NoGo trials. We observed a significant “condition × time” interaction in behavioral performance (mean reaction time, F(8,120) = 2.4, P<0.05; hit rates, F(8,120) = 10.8, P<0.01; false alarm rate, F(8,120) = 12.6, P<0.01); however there is not a significant difference between the zaleplon condition and the placebo condition. Mean reaction time (F(2,30) = 0.25, P>0.05), hit rates (F(2,30) = 0.07, P>0.05) and false alarm rates (F(2,30) = 0.30, P>0.05) were not different among the three conditions at baseline. Mean reaction times became markedly slower after 30 h of total sleep deprivation (zaleplon vs. placebo, P>0.05; zaleplon vs. control, P = 0.04; placebo vs. control, P = 0.02) and sudden awakening (zaleplon vs. placebo, P>0.05; zaleplon vs. control, P<0.01; placebo vs. control, P<0.01) from the nap when compared to the control condition but then returned to normal level. The hit rates after were significantly reduced, and false alarm rates increased after TSD (hit rates, zaleplon vs. placebo, P>0.05; zaleplon vs. control, P<0.01; placebo vs. control, P<0.01; false alarm rates, zaleplon vs. placebo, P>0.05; zaleplon vs. control, P<0.01; placebo vs. control, P<0.01) and sudden awakening (hit rates, zaleplon vs. placebo, P>0.05; zaleplon vs. control, P<0.01; placebo vs. control, P<0.01; false alarm rates, zaleplon vs. placebo, P = 0.04; zaleplon vs. control, P<0.01; placebo vs. control, P<0.01) compared to the control condition. Although the subjects performed markedly better at 4 h (hit rates, zaleplon vs. placebo, P>0.05; zaleplon vs. control, P<0.01; placebo vs. control, P<0.01; false alarm rates, zaleplon vs. placebo, P>0.05; zaleplon vs. control, P<0.01; placebo vs. control, P<0.01) and 6 h post-drug(hit rates, zaleplon vs. placebo, P>0.05; zaleplon vs. control, P = 0.03; placebo vs. control, P = 0.03; false alarm rates, zaleplon vs. placebo, P>0.05; zaleplon vs. control, P<0.01; placebo vs. control, P<0.01), hit rates and false alarm rates nevertheless demonstrate differences between the two treatment conditions and the control condition at all time points.

**Table 1:** Mean reaction time (ms), hit rates in the Go trials and false alarm rates in the NoGo trials.

		Baseline	SD 30h	Post-drug	Post-drug	Post-drug
				2h	4h	6h
Mean reaction time(ms)	Zaleplon	336(28)	364(44)	370(32)	346(32)	348(29)
	Growth rate	−1%	11%	14%	4%	6%
	Placebo	332(21)	366(31)	371(28)	350(39)	354(35)
	Growth rate	−2%	12%	14%	5%	8%
	Control	338(25)	328(24)	325(22)	332(27)	329(29)
Hit rates	Zaleplon	0.98(0.01)	0.96(0.02)	0.93(0.03)	0.97(0.02)	0.96(0.02)
	Growth rate	0%	−3%	−6%	−2%	−2%
	Placebo	0.98(0.01)	0.96(0.02)	0.93(0.03)	0.97(0.02)	0.97(0.02)
	Growth rate	0%	−3%	−6%	−2%	−1%
	Control	0.98(0.01)	0.99(0.01)	0.99(0.01)	0.99(0.01)	0.98(0.01)
False alarms	Zaleplon	0.08(0.02)	0.17(0.04)	0.19(0.05)	0.13(0.04)	0.12(0.03)
	Growth rate	14%	183%	280%	117%	100%
	Placebo	0.07(0.03)	0.18(0.04)	0.24(0.08)	0.12(0.04)	0.14(0.04)
	Growth rate	0%	200%	380%	100%	133%
	Control	0.07(0.03)	0.06(0.04)	0.05(0.03)	0.06(0.02)	0.06(0.03)

Note: The growth rate represents the growth rate of absolute value.

### NoGo-P3

**Figure 3:**
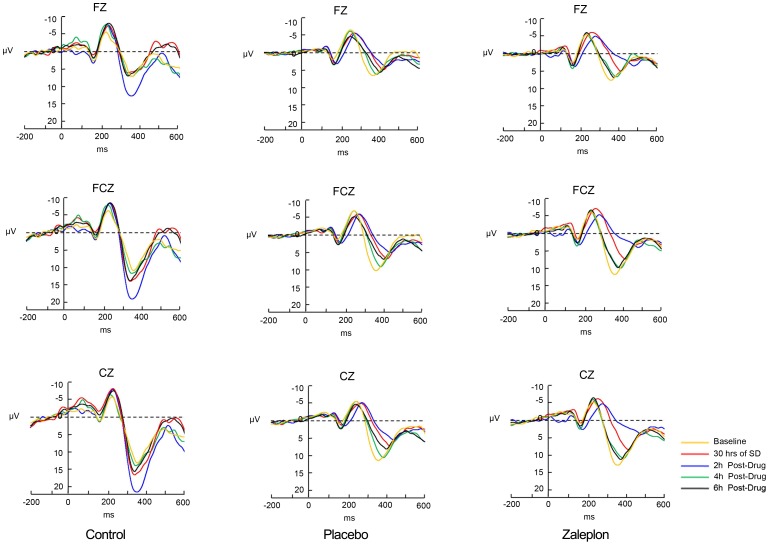
Grand-average ERPs at Fz, Cz, and Pz for NoGo trials at five different time points in zaleplon, placebo and control groups.

Fig. 3 shows the grand-average ERPs at the Fz, FCz, and Cz sites for the NoGo trials in the zaleplon condition, placebo condition and control condition, respectively. The means and standard deviations of NoGo-P3 latencies and amplitudes are shown in Table 2. A significant “condition × time” interaction was detected for NoGo-P3 latencies at Fz (F(8,120) = 49.63, P<0.01), FCz (F(8, 120) = 53.71, P<0.01) and Cz (F(8, 120) = 32.23, P<0.01) sites. The interaction effect was because participants had longer NoGo-P3 latency in zaleplon condition at 2 h post-drug but a shorter NoGo-P3 latency at 4 h and 6 h post-drug in the zaleplon condition. This interaction was resolved by analyzing the simple effects by fixing the five time points, respectively. At the FCz site, for example, at first, there was no difference among the three conditions at baseline (F(2,30) = 0.64, P>0.05). However, the latencies of zaleplon and the placebo markedly increased by 24% and 26%, respectively, more than the control condition after 30 h of SD (zaleplon vs. placebo, P>0.05; zaleplon vs. control, P<0.01; placebo vs. control, P<0.01). Then, we observed that the latency of the zaleplon condition increased by 46%, whereas the latency of the placebo increased by 36% over the control condition at sudden awakening (zaleplon vs. placebo, P<0.01; zaleplon vs. control, P<0.01; placebo vs. control, P<0.01). In addition, the growth rate of NoGo-P3 latency in the zaleplon condition was significantly higher than in the placebo condition according to simple effects analysis. However, the latency of zaleplon increased by 9% over the control, whereas the latency of placebo increased by 19% over the control at 6 h post drug (zaleplon vs. placebo, P<0.01; zaleplon vs. control, P = 0.04; placebo vs. control, P<0.01). Thus, the growth rate of the latency of the zaleplon was markedly lower than placebo at the last time point which was opposite to the result of the third time point - sudden awakening.

**Table 2:** Latency (ms) and amplitude (microvolts) of NoGo-P3 at Fz, Cz, and Pz.

		Baseline	SD 30h	Post-drug	Post-drug	Post-drug
				2h	4h	6h
Latency						
Fz	Zaleplon	376(26)	438(31)	492(31)	390(30)	395(35)
	Growth rate	1%	28%	46%	7%	12%
	Placebo	368(26)	439(27)	460(35)	404(33)	423(34)
	Growth rate	−1%	29%	36%	11%	20%
	Control	373(32)	341(23)	337(24)	363(23)	352(26)
FCz	Zaleplon	364(26)	426(27)	490(34)	388(31)	384(32)
	Growth rate	−2%	24%	45%	7%	9%
	Placebo	361(27)	431(33)	459(35)	398(26)	418(31)
	Growth rate	−2%	26%	36%	10%	19%
	Control	370(28)	343(26)	338(23)	361(23)	351(24)
Cz	Zaleplon	365(27)	430(33)	468(31)	385(32)	382(24)
	Growth rate	0%	25%	38%	7%	9%
	Placebo	372(24)	427(34)	460(37)	395(29)	410(25)
	Growth rate	2%	24%	35%	10%	16%
	Control	366(31)	344(23)	340(24)	359(27)	352(26)
Amplitude						
Fz	Zaleplon	8.2(3.4)	5.4(2.7)	4.4(2.2)	7.1(3.4)	6.9(2.8)
	Growth rate	11%	−27%	−56%	−7%	−1%
	Placebo	7.2(3.5)	5.1(2.9)	4.8(2.9)	6.4(3.4)	6.2(3.3)
	Growth rate	−3%	−31%	−52%	−16%	−11%
	Control	7.4(3.7)	7.4(3.0)	9.9(3.3)	7.6(3.5)	7.0(3.3)
FCz	Zaleplon	12.3(5.2)	7.6(3.4)	4.9(2.2)	10.5(4.4)	10.0(3.6)
	Growth rate	10%	−50%	−70%	−15%	−28%
	Placebo	11.0(3.9)	7.1(3.1)	5.9(2.9)	9.2(3.4)	7.6(3.3)
	Growth rate	−2%	−53%	−64%	−26%	−45%
	Control	11.2(4.0)	15.1(3.6)	16.6(4.0)	12.4(4.7)	13.9(3.9)
Cz	Zaleplon	13.6(4.5)	8.6(3.1)	4.7(2.5)	11.7(4.6)	11.1(4.8)
	Growth rate	1%	−51%	−76%	−19%	−29%
	Placebo	12.2(5.4)	7.3(3.3)	6.2(3.4)	10.7(5.0)	9.1(5.0)
	Growth rate	−9%	−59%	−69%	−26%	−42%
	Control	13.4(3.5)	17.6(4.3)	19.7(6.2)	14.5(5.5)	15.7(5.7)

Note: The growth rate represents the growth rate of absolute value.

A significant “condition × time” interaction was also detected for NoGo-P3 amplitudes at Fz(F(8, 120) = 4.45, P<0.01), FCz(F(8, 120) = 13.86, P<0.01) and Cz(F(8, 120) = 13.93, P<0.01) sites. This interaction was similar as NoGo-P3 latencies.

### NoGo-N2

The means and standard deviations of NoGo-N2 latencies and amplitudes are shown in Table 3. A significant “condition × time” interaction was observed for NoGo-N2 latencies at Fz(F(8, 120) = 4.37, P<0.01), FCz(F(8, 120) = 3.89, P<0.01) and Cz(F(8, 120) = 4.97, P<0.01) sites. There was also no difference among the three conditions at baseline (F(2,30) = 0.92, P>0.5). The latencies slightly increased by 9% and 8% in the zaleplon and placebo conditions, respectively, after 30 h of SD (zaleplon vs. placebo, P>0.05; zaleplon vs. control, P>0.05; placebo vs. control, P>0.05), but the differences are not significant. Subsequently, we observed both of the zaleplon (19%) and placebo (14%) conditions were significantly more prolonged than control at sudden awakening (zaleplon vs. placebo, P>0.05; zaleplon vs. control, P<0.01; placebo vs. control, P = 0.01). Although both zaleplon and placebo latencies became shorter at 4 h post-drug, there was nevertheless a difference between the placebo and control conditions (zaleplon vs. placebo, P>0.05; zaleplon vs. control, P>0.05; placebo vs. control, P = 0.02). At the last time point, there was no difference between the zaleplon and placebo conditions and the control condition (zaleplon vs. placebo, P>0.05; zaleplon vs. control, P>0.05; placebo vs. control, P>0.05).

**Table 3:** Latency (ms) and amplitude (microvolts) of NoGo-N2 at Fz, Cz, and Pz.

		Baseline	SD 30h	Post-drug	Post-drug	Post-drug
				2h	4h	6h
Latency						
Fz	Zaleplon	254(26)	276(26)	296(27)	253(29)	252(23)
	Growth rate	4%	9%	21%	9%	1%
	Placebo	255(21)	277(23)	287(23)	258(27)	264(26)
	Growth rate	5%	9%	17%	11%	6%
	Control	244(31)	253(28)	245(27)	233(27)	250(29)
						
FCz	Zaleplon	250(22)	273(24)	291(28)	249(25)	246(23)
	Growth rate	2%	9%	19%	9%	−1%
	Placebo	257(24)	271(28)	279(29)	255(24)	262(28)
	Growth rate	5%	8%	14%	12%	5%
	Control	245(26)	251(24)	244(21)	228(19)	250(25)
Cz	Zaleplon	243(22)	261(26)	287(29)	242(24)	243(21)
	Growth rate	1%	10%	21%	9%	1%
	Placebo	247(20)	265(28)	271(26)	251(23)	254(25)
	Growth rate	2%	11%	14%	13%	6%
	Control	241(24)	238(23)	238(21)	223(19)	240(21)
Amplitude						
Fz	Zaleplon	−6.9(2.9)	−7.3(3.4)	−6.3(2.5)	−7.0(3.1)	−6.8(3.0)
	Growth rate	−1%	−12%	−22%	−18%	−23%
	Placebo	−7.2(3.4)	−7.1(3.4)	−6.7(2.9)	−7.0(4.1)	−6.4(3.2)
	Growth rate	3%	−14%	−17%	−18%	−27%
	Control	−7.0(3.1)	−8.3(2.7)	−8.1(3.2)	−8.5(2.9)	−8.8(3.4)
FCz	Zaleplon	−7.4(2.8)	−8.2(3.2)	−6.8(2.8)	−7.8(3.5)	−7.6(3.6)
	Growth rate	−10%	−9%	−29%	−13%	−16%
	Placebo	−7.7(3.6)	−7.4(3.9)	−7.3(3.2)	−7.8(3.0)	−7.1(3.8)
	Growth rate	−6%	−18%	−24%	−13%	−21%
	Control	−8.2(2.5)	−9.0(2.9)	−9.6(3.3)	−9.0(3.5)	−9.0(3.1)
Cz	Zaleplon	−6.4(2.9)	−7.5(2.6)	−6.4(2.8)	−7.3(3.0)	−7.4(3.7)
	Growth rate	−17%	−11%	−37%	−1%	−5%
	Placebo	−6.6(3.6)	−6.7(3.7)	−6.7(3.5)	−6.6(3.0)	−6.6(3.4)
	Growth rate	−14%	−20%	−34%	−11%	−15%
	Control	−7.7(3.5)	−8.4(3.5)	−10.1(3.1)	−7.4(2.2)	−7.8(2.5)

Note: The growth rate represents the growth rate of absolute value.

No effect was observed for NoGo-N2 amplitudes at any sites.

## Discussion

In the present study, we used a Go/NoGo task to evaluate the effect of a zaleplon-induced nap as a countermeasure to sleep deprivation and cognitive decline by focusing on NoGo-N2 and NoGo-P3 components connected with impulse inhibition.

In the behavioral performances, although we observed an interaction effect, there was no significant difference after analysis between the zaleplon condition and the placebo However, because the index used in behavioral performance is false alarm rate; the result may only generally reflect the level of impulse inhibition function. Because impulse inhibition is a complex process, the behavioral results in the present study may not sensitively reflect some important details as such as ERP results. In ERP results, the N2 and P3 components could reflect two different sub-processes in impulse inhibition.

As the ERP results indicated, there is no difference among the zaleplon, the placebo and the control conditions at baseline. Additionally, we observed a significant increase in NoGo-P3 peak latencies and reduction in P3 amplitudes after 30 h of SD in two treatment conditions compared with the control condition. These findings show that SD affects both stimulus evaluation time and controlled processing for response inhibition. The effect reported in this article is consistent with previous studies. Higushi and colleagues observed that there is a positive correlation between the latency of P300 and sleepiness during the day [28]. Several researchers have observed prolonged P3 latency and reduced amplitude during extended wakefulness [24], [29], [30], [31]. The amplitude of the P300 component of the ERPs is relevant to the deployment of attentional resources. In addition, its latency reflects the time required for stimulus categorization and evaluation. Sleep loss results in subjects' having difficulty allocating resources to detect NoGo stimuli. We also observed that NoGo-N2 latencies of the zaleplon and placebo conditions had the identical tendency as NoGo-P3. NoGo-N2 amplitude was not modulated. One possible explanation was that N2 amplitude covaried with the magnitude of false alarm rates [32] because false alarm rates increased after sleep deprivation. Another explanation could be that an increased cerebral compensatory response increased monitoring demand in response selection caused by sleep deprivation [33]. The increase in reaction time after sleep deprivation was relevant to changes in the speed of response-selection processes, which was reflected in the prolonged NoGo-N2 latency following sleep deprivation.

At sudden awakening, we observed a marked increase in NoGo-P3 and NoGo-N2 latencies and a significant decrease in NoGo-P3 amplitude in both the zaleplon condition and placebo condition compared with the control condition. An important consideration in the use of a nap, which is used as a countermeasure, is sleep inertia. Sleep inertia is a transitional state of lowered arousal occurring immediately after awakening from sleep and producing a temporary reduction in subsequent performance, including short-term, memory, vigilance and other measures of cognitive functioning as well as reaction time, ability to resist sleep and grip strength [34]. Generally speaking, performance will be lowest during the first 5 min following awakening; however, it generally recovers after 15 to 30 min [35]. Perhaps the adverse effects of sleep deprivation on sleep inertia magnitude should be avoided by any person who may have to perform important tasks immediately after awakening. In addition to the above-mentioned, the latencies of the zaleplon condition were longer, and the amplitudes were observed, by simple effect analysis, to be lower than the placebo amplitude. At this point, zaleplon had decayed to half its full concentration. We concluded that conflict suppression ability may be negatively affected by zaleplon, which lasted for at least 2 h post-drug. Jeffrey and his colleagues suggested that zaleplon may have a differential effect on levels of cognition and ability using only behavioral data [36]. Jeffrey and his colleagues inferred that basic cognitive performance (i.e., simple reaction time) and basic physical performance (i.e., grip strength) after awakening from a short zaleplon-induced nap may not be negatively affected to any great extent. Nevertheless, higher cognitive performance (e.g., mathematical processing) appears to suffer greatly. In the study, we provide evidence of zaleplon effects on inhibition functioning by ERPs. In brief, when naps are possible but the time available is not sufficient, zaleplon diminishes the time to sleep onset and results in more time asleep during a nap period of restricted duration. However, to reduce problems, individuals planning to take zaleplon should place themselves in a safe environment in which performance is not strongly demanded and allow enough time after awakening to avoid sleep inertia and side effects, approximately 2 h after a 10 mg dose.

NoGo-P3 latency at Cz and NoGo-N2 latencies at FCz and Cz in the zaleplon condition became shorter and were no different from the control condition at 4 h post-drug. Meanwhile, both amplitudes of NoGo-P3 in the zaleplon and placebo conditions increased and were not different from the control condition at all sites. Therefore, we obtained results consistent with most previous articles: naps from 1 h to 8 h enhance performance and alertness during continuous operations[19]. For instance, in a study by Naitoh and his colleagues, subjects were given a 3-h nap after being awake for approximately 24 h; however, they were then required to stay awake for an additional 20 h. The results showed that this 3-h nap decreased the decline in performance during the extra work period [37].

NoGo-P3 latencies at Fz and FCz of the placebo condition showed a marked increase at 6 h post-drug compared with 4 h post-drug when the condition was fixed; performance, however, was much better than after 30-h SD and sudden awakening at these two time points. In another manner, NoGo-P3 latencies demonstrated no difference between the zaleplon and placebo conditions at 4 h post-drug with a fixed time point; however, they were significantly delayed in the placebo condition at 6 h post-drug, leading to differences between the two groups. However, ERP data remained stable in the zaleplon condition at these two time points. The identical tendency occurred in the amplitude of NoGo-N2 at the FCz and Cz sites. The difference between the placebo and control conditions appeared at 6 h post-drug although the difference did not exist at 4 h post-drug. This result revealed that cognitive function of participants demonstrated a better recovery from the zaleplon-induced nap than the placebo-induced nap. This indicated that subjects were more capable of maintaining wakefulness after a zaleplon-induced nap than a placebo-induced nap throughout the testing day, and the zaleplon nap was better than the placebo nap at 4 h and 6 h post-drug time points with the exception of sudden awakening.

Although the findings in the present study are meaningful, some limitations must be addressed. For technical reasons, the EEG recordings during the naps in the present study were not available. If this part of the data could be analyzed, we could learn how the sleep structure changed during the drug-induced nap. The conclusions of the present study would thus be more credible. Additionally, the sample size in the present study was small and, all the subjects were male. Thus, we must be cautious when discussing our findings. The sample size should be increased, and female subjects should be included in further studies.

## Conclusions

Zaleplon can be quite beneficial in situations in which there is only a brief period available for sleep because of its rapid absorption. When personnel have only 2 h for a nap, zaleplon can ensure better performance maintenance after awakening. Subjects were better at conflict detection and response inhibition after using a zaleplon-induced nap as a countermeasure to sleep deprivation with the NoGo-N2 and NoGo-P3 components of ERPs as indices. After, a zaleplon-induced nap, managers must take care to avoid the subsequent problems connected with post-nap sleep inertia by allowing personnel enough time to completely awaken from naps before returning to work. In addition, when zaleplon is used to initiate a 2-h prophylactic nap, one should be vigilant in monitoring the medication's side effects for at least 2 h post-drug.
